# Methods and criteria for validating the multimodal functions of perinatal derivatives when used in oncological and antimicrobial applications

**DOI:** 10.3389/fbioe.2022.958669

**Published:** 2022-10-13

**Authors:** Antonietta R. Silini, Taja Železnik Ramuta, Ana Salomé Pires, Asmita Banerjee, Marie Dubus, Florelle Gindraux, Halima Kerdjoudj, Justinas Maciulatis, Adelheid Weidinger, Susanne Wolbank, Günther Eissner, Bernd Giebel, Michela Pozzobon, Ornella Parolini, Mateja Erdani Kreft

**Affiliations:** ^1^ Centro di Ricerca E. Menni, Fondazione Poliambulanza Istituto Ospedaliero, Brescia, Italy; ^2^ Faculty of Medicine, Institute of Cell Biology, University of Ljubljana, Ljubljana, Slovenia; ^3^ Faculty of Medicine, Coimbra Institute for Clinical and Biomedical Research (iCBR) Area of Environment, Genetics and Oncobiology (CIMAGO), Institute of Biophysics, University of Coimbra, Coimbra, Portugal; ^4^ Center for Innovative Biomedicine and Biotechnology (CIBB), University of Coimbra, Coimbra, Portugal; ^5^ Clinical Academic Center of Coimbra (CACC), Coimbra, Portugal; ^6^ Ludwig Boltzmann Institute for Traumatology, The Research Center in Cooperation with AUVA, Austrian Cluster for Tissue Regeneration, Vienna, Austria; ^7^ Université de Reims Champagne Ardenne, EA 4691 Biomatériaux et Inflammation en Site Osseux (BIOS), Reims, France; ^8^ Service de Chirurgie Orthopédique, Traumatologique et Plastique, CHU Besançon and Laboratoire de Nanomédecine, Imagerie, Thérapeutique EA 4662, Université Bourgogne Franche-Comté, Besançon, France; ^9^ The Institute of Physiology and Pharmacology, Medical Academy, Lithuanian University of Health Sciences, Kaunas, Lithuania; ^10^ Systems Biology Ireland, UCD School of Medicine, University College Dublin, Dublin, Ireland; ^11^ Institute for Transfusion Medicine, University Hospital Essen, University of Duisburg-Essen, Essen, Germany; ^12^ Stem Cells and Regenerative Medicine Lab, Department of Women’s and Children’s Health, University of Padova, Fondazione Istituto di Ricerca Pediatrica Città Della Speranza, Padoa, Italy; ^13^ Department of Life Science and Public Health, Università Cattolica Del Sacro Cuore, Rome, Italy; ^14^ Fondazione Policlinico Universitario “Agostino Gemelli” IRCCS, Rome, Italy

**Keywords:** perinatal derivatives, biological assays, functional assays, potency assays, mechanisms of action, pharmacologic actions, cancer, infections

## Abstract

Perinatal derivatives or PnDs refer to tissues, cells and secretomes from perinatal, or birth-associated tissues. In the past 2 decades PnDs have been highly investigated for their multimodal mechanisms of action that have been exploited in various disease settings, including in different cancers and infections. Indeed, there is growing evidence that PnDs possess anticancer and antimicrobial activities, but an urgent issue that needs to be addressed is the reproducible evaluation of efficacy, both *in vitro* and *in vivo*. Herein we present the most commonly used functional assays for the assessment of antitumor and antimicrobial properties of PnDs, and we discuss their advantages and disadvantages in assessing the functionality. This review is part of a quadrinomial series on functional assays for the validation of PnDs spanning biological functions such as immunomodulation, anticancer and antimicrobial, wound healing, and regeneration.

## 1 Introduction

Perinatal derivatives or PnDs (that include tissues and cells and secretomes from perinatal, or birth-associated tissues), and especially mesenchymal stromal cells (MSC) from perinatal tissues, have been mostly exploited for their applications in regenerative medicine, substantiated by their ability to modulate immune responses and/or to act in a pro-regenerative manner by promoting progenitor/stem cell differentiation (Silini et al., 2017; Silini et al., 2019). What has been largely unexplored is the applications of PnDs in other sectors, such as in oncology.

As a matter of fact, PnDs have only recently been investigated *in vitro* and *in vivo* for their potential as antitumor therapies. In this context, PnDs have been shown to act multimodally through complex mechanisms of action (MoA), and to target various hallmarks of cancer that capture essential features of tumorigenesis, such as sustaining proliferative signaling, evading growth suppressors, resisting cell death, enabling replicative immortality, inducing angiogenesis, activating invasion and metastasis, reprogramming cellular metabolism, avoiding immune destruction, tumor-promoting inflammation, genome instability, and deregulating cellular metabolism (Hanahan and Weinberg 2011). Treatments that shift the focus to the systemic level and that target the hallmarks of cancer are urgently needed and highly relevant.

In the context of cancer, PnDs have been reported to play opposing roles. Herein we will discuss these studies along with the *in vitro* and *in vivo* assays implemented to obtain a clearer understanding of the potential applications of PnDs as an antitumor treatment.

In addition to the potential applications of PnDs in oncology, several PnDs have been shown to possess antimicrobial properties (King et al., 2007a; Klaffenbach et al., 2011; Ramuta et al., 2021a), which is highly relevant in the field of oncology. As a matter of fact, in the last decade, growing evidence has emerged demonstrating that microbiota and microbial pathogens have immense effect on cancer development and treatment (Bhatt et al., 2017; Raza et al., 2019; Jain et al., 2021). While in 15–20% of cancer cases microbial pathogens drive tumorigenesis, even a larger part of malignancies is associated with the altered composition of commensal microbiota (Bhatt et al., 2017). Furthermore, bacteria are also able to affect the efficacy of chemotherapeutic drugs, either by inhibiting or enhancing their effect (Lehouritis et al., 2015; Roy and Trinchieri 2017). Namely, the microbiota and pathogens may affect the pharmacokinetics, antitumor activity and drug toxicity of chemotherapeutic agents by 1) changing the chemical structure of the drug (biotransformation) (Haiser and Turnbaugh 2012; Wilson and Nicholson 2017), 2) decreasing the absorption of certain drugs (Carmody and Turnbaugh 2014), or even indirectly by 3) affecting the host’s gene expression and physiology (e.g., of the local mucosal barrier), which results in altered metabolism of drugs (Björkholm et al., 2009; Selwyn et al., 2015; Selwyn et al., 2016; Roy and Trinchieri 2017).

Thus, herein both the anticancer and antimicrobial properties will be discussed alongside one another to provide a better understanding of the potential applications of PnDs as therapeutic strategies in these fields. We briefly summarize the results obtained using PnDs and then focus on the most frequently used functional assays for analyzing the antitumor and antimicrobial effects of PnDs in order to potentially provide insight into the future development of new functional and/or potency assays.

This review is part of a series of contributions from the COST Action (CA17116) entitled “International Network for Translating Research on Perinatal Derivatives into Therapeutic Approaches-SPRINT”. This Action is broadly aimed at establishing consensus for different aspects of PnDs research. The aim of this review is to provide inputs for the development of functional and potency assays that can be used to test PnDs before their oncological and antimicrobial application.

## 2 The effects of perinatal derivatives on the hallmarks of cancer

In this section we summarize the most frequently used *in vitro* and *in vivo* assays for analyzing the effects of PnDs on several hallmarks of cancer such as cell proliferation and metabolism, cell death and apoptosis, cell migration and invasion, angiogenesis, and metastasis.

### 2.1 Investigating perinatal derivatives actions on tumor cell proliferation

One of the most widely analyzed hallmark of tumor cells is proliferation. Indeed, *in vitro* PnDs have been shown to mostly exert antiproliferative effects on tumor cells (Gauthaman et al., 2012; Magatti et al., 2012; Liu et al., 2013; Hendijani et al., 2015; Kalamegam et al., 2019; Ramuta et al., 2020a), but it has also been shown that they may induce tumor cell proliferation (Kim et al., 2015; Li et al., 2015).

Several assays are used to assess the effect of PnDs on tumor cell proliferation. The investigation of DNA synthesis analyzes the effects of PnDs on tumor cell proliferation. Incorporation of radiolabeled DNA precursor 3H-thymidine (Ayuzawa et al., 2009; Magatti et al., 2012; Marleau et al., 2012; Yuan et al., 2013; Riedel et al., 2019; Ramuta et al., 2020b) into new strands of chromosomal DNA or more recent analogs, BrdU (bromoeoxyuridin) (Tian et al., 2010; Gauthaman et al., 2012; Han et al., 2014; Hendijani et al., 2015) and EdU (5-ethynyl-2 deoxyuridine) (Wang M et al., 2015; Janev et al., 2021), have been widely used to assess *de novo* DNA synthesis. 3H-thymidine detection requires radioactive labeling followed by detection with a scintillation beta-counter, while BrdU can be easily detected by antibodies followed by flow cytometry or by immunohistochemistry. BrdU is mutagenic and can alter the cell cycle, thus adequate controls should be implemented. In contrast to BrdU, the newer analog EdU does not require DNA denaturation by exposing cells to HCl, heat or DNAse, and its detection is rapid and highly sensitive. BrdU and EdU are considered reliable assays for a direct index of proliferation. Other analyses implemented for the analysis of tumor cell proliferation are Ki67 cell immunolabelling (Lin et al., 2014; Riedel et al., 2019), and other assays aimed at investigating genes or proteins involved in cell cycle progression, such as cyclins (Magatti, De Munari et al., 2012; Riedel, Pérez-Pérez et al., 2019).

The colony forming unit (CFU) assay analyzes the consequences of external stress signals on the cell’s ability to proliferate and form a colony. This assay has been widely used to test the effect of PnDs (Ayuzawa et al., 2009; Liu et al., 2013; Ciavarella et al., 2015; Li et al., 2015; Wang M et al., 2015; Wang W et al., 2015) and is especially useful to assess long-term effects. This method is however time consuming with extended incubation times, plus colony formation ability differs between cells. The traditional assay requires weeks for completion and usually does not allow cell retrieval; however, this can be circumvented by the use of fluorescent dyes allowing quantitative, high throughput colony counting and by the use of specialized agars that allow cell suspension and growth. The assay can also be used to predict tumorigenicity *in vivo*.

*In vivo* objective assessment of tumor growth is a crucial tool for the advancement of cancer therapies. *In vivo*, PnDs have been shown to both inhibit (Ayuzawa et al., 2009; Wu et al., 2013) and induce (Yang C. et al., 2014; Wang M et al., 2015; Svitina et al., 2018) tumor growth. These contradictory results could be due to several variations, either given by the PnDs used (e.g., tissues, cells, secretomes, homogenates), by the tumor model (e.g., tumor cells, rodent model and strain), by the different treatment regimens (e.g., administration route, dosage), or by the method used to monitor tumor progression (e.g., caliper measurements, imaging).

Monitoring tumor progression after PnDs treatment has been performed through the measurement of tumor diameters (Du et al., 2014; Ma et al., 2015; Bu et al., 2017; Yuan L et al., 2019) (or volumes deduced from diameters) or tumor weight, but also through more sophisticated *in vivo* optical imaging techniques (Di et al., 2014; Leng et al., 2014; Ciavarella et al., 2015; Cafforio et al., 2017; Zhang et al., 2017; Yuan Z et al., 2019; Chetty et al., 2020), which include fluorescence and bioluminescence studies. Although traditional caliper measurement is a simple and low-cost method, its major disadvantages include variability of tumor size measurements and tumoral heterogeneity. In contrast, optical imaging is a more accurate, sensitive and specific technique for tumor imaging, allowing the detection of microscopic tumors. The main limitation of optical imaging is the need of tumor cells to express a reporter gene (Puaux et al., 2011). Cell proliferation is often used as a measure of tumor response, with immunohistochemistry being the most widely used *in vivo* technique.

### 2.2 Investigating perinatal derivatives actions on tumor cell metabolism

Altered cell metabolism is another feature of tumor cells, and proliferating tumor cells hijack their metabolism to fuel continuous growth. Cell metabolism is commonly measured through the reduction of substrates to a final product by intracellular enzyme activity in living cells can be assessed by colorimetric assays. The degree of color change is not directly proportional to the number of viable cells, but rather to enzyme activity. This method permits only a moderately robust measurement of viability; however, the ease of use and potential for high throughput analysis in multiwell plates has made it very popular. The most frequently used tetrazolium compound (MTT) is reduced to formazan (Li et al., 2011; Chao et al., 2012; Gauthaman et al., 2012; Kang et al., 2012; Liu et al., 2013; Lin et al., 2014; Niknejad et al., 2014; Rolfo et al., 2014; Bonomi et al., 2015; Lang et al., 2015; Li et al., 2015; Kamalabadi-Farahani et al., 2018; Mandal et al., 2019; Riedel et al., 2019). A less toxic alternative, Cell Counting Kit-8 (CCK-8) (Wu et al., 2013; Yan et al., 2013; Yang C. et al., 2014; Kim et al., 2015; Di Germanio et al., 2016), has also been used, with a detection sensitivity higher than tetrazolium salts such as MTT or MTS(Wang W et al., 2015). Tumor cell oxidative stress, using superoxide dismutase, intracellular accumulation of reactive oxygen species (ROS), glutathione peroxidase, hydrogen peroxide and lipid peroxidation assays have also been investigated after PnDs treatment (Lin et al., 2014).

### 2.3 Investigating perinatal derivatives actions on tumor cell death and apoptosis

Similar to their effects on tumor cell proliferation PnDs have been shown to have dual effects on tumor cell death and apoptosis, by either promoting (Chen et al., 2012; Jiao et al., 2012; Del Fattore et al., 2015; Kalamegam et al., 2018) or inhibiting (Niknejad et al., 2014) these processes.

There are several assays used to assess the effect of PnDs on tumor cell death. For example, annexin V/PI assay and flow cytometry are the most popular approaches for detection of apoptosis in tumor cells after PnDs treatment (Gauthaman et al., 2012; Wu et al., 2013; Yang X. et al., 2014; Niknejad et al., 2014; Mamede et al., 2015; Lin et al., 2016; Paris et al., 2016; Shen et al., 2016; Lin H et al., 2017; Chai et al., 2018; Jiao et al., 2018; Yuan et al., 2018; Khalil et al., 2019; Rezaei-Tazangi et al., 2020; Silva et al., 2020). The TUNEL assay (Wu et al., 2013; Niknejad et al., 2014) detects DNA fragmentation in apoptotic cells *in vitro*. After staining, cells can be analyzed by light or fluorescent microscopy. TUNEL staining is fast, accurate and sensitive but fails in discriminating the different types of cell death. The assay can also be used to assess the effect of PnDs on tumor cell death *ex vivo*. Detection of apoptosis regulators, such as caspases (Wu et al., 2013; Yang C. et al., 2014; Niknejad et al., 2014; Mamede et al., 2015; Lin et al., 2016; Kalamegam et al., 2018; Mandal et al., 2019; Rezaei-Tazangi et al., 2020), cytochrome c, Bcl-2, Bax, Fas, FasL, Danger Associated Molecular Proteins (DAMPs), CRT, Hsp90 and Hsp70, etc. Can be detected at the protein and/or mRNA level using flow cytometry, multi-detection plate reader (Mamede et al., 2015), immunoblot (Dzobo et al., 2016; Shen et al., 2016; Yuan et al., 2018), immunohistochemistry, immunofluorescence (Dzobo et al., 2016; Lin H et al., 2017), and RT-PCR (Yang X. et al., 2014). As further progress is made in understanding the mechanisms of cell death, more accurate and precise interpretations of the results of these tests will be possible.

Cell death induced by PnDs in animal models has been mainly analyzed by *in situ* detection of apoptosis as mentioned previously regarding the *in vitro* assays (Dong et al., 2018; Kamalabadi-Farahani et al., 2018; Chen et al., 2019; Fan et al., 2020). An alternative approach is the *in vivo* imaging of apoptosis, using radiolabeled forms of annexin V for positron emission tomography (PET) and single photon emission computed tomography (SPECT) (Iravani and Hicks 2020).

Mitochondria play a key role in response to cellular stress and injury. For this reason, the evaluation of mitochondrial membrane potential (MMP) (Mamede et al., 2015; Lin et al., 2016) could also be used as the marker of cell death. However, the interpretation should be carefully and critically performed. Measurement of mitochondrial activity could not only serve as an alternative to cell viability assays, but also provide distinct information on the metabolic state, and therefore the quality of a cell. For the application of therapeutic cells, knowledge on the mode of energy production can be important. Mitochondrial activity can be determined by quantification of the activity of single complexes such as complex I by measuring the light absorbance of nicotinamide adenine dinucleotide (NADH), the electron donor for complex I, at 340 nm (Nelson and Cox 2004). The downside of this method is that it does not provide any information on the coupling state of the electron transfer system, and therefore, also not on the production level of adenosine triphosphate (ATP). Activity of the entire electron transfer system can be determined, for example, with a Clark electrode-based measurement (Hütter et al., 2006). With this method, the oxygen concentration of a solution is measured as oxygen is reduced at the cathode. The resulting current is directly proportional to the oxygen concentration of the solution (Gnaiger 2008). The advantage of this method is that distinct respiration states can be determined (Gnaiger 2008). By addition of specific substrates and inhibitors, total oxygen consumption (routine respiration) can be distinguished from oxygen consumption with ATP production (oxidative phosphorylation) and oxygen consumption without ATP production (LEAK) (Gnaiger 2008). With this method, mitochondrial respiration can be measured in tissue, tissue homogenate, cells and isolated mitochondria. To our knowledge, there are no studies that have evaluated the effects of PnD on tumor MMP, however, such measurements have successfully been performed in human amniotic membrane tissue (Banerjee et al., 2015; Poženel et al., 2019), isolated human amniotic membrane epithelial cells (Banerjee et al., 2018a), and human amniotic membrane derived MSC (Banerjee et al., 2018a; Banerjee et al., 2018b). In addition to the mitochondrial respiration assay, mitochondrial status can also be monitored with the membrane permeable dye JC-1. JC-1 is a fluorescent cationic carbocyanine dye that exhibits potential-dependent accumulation in mitochondria, forming J-aggregates and diffuses across mitochondria upon depolarisation to form a monomeric state (Sivandzade et al., 2019). Currently, to our knowledge, there is only one study that has investigated the function of amniotic membrane proteins (AMPs) extracted from hAM against hypoxia-induced H9c2 cardiomyoblast cells (Faridvand et al., 2019). AMPs have potent cardioprotective effects in H9c2 cells by inhibiting the Ca2+ overload and the mitochondrial membrane potential dysfunction during hypoxia (Faridvand et al., 2019). Furthermore, there are few studies that evaluated mitochondrial membrane potential in placental trophoblast cells from patients with preeclampsia (Zhang et al., 2022). The anticancer effect of PnDs have not yet been evaluated with the mitochondrial dye JC-1.

In this section, we presented an overview of the most common functional assays that have been used for evaluating the pro-cell death/proapoptotic activity of PnDs. Each assay has its advantages and an understanding of strengths and limitations can allow for the selection of the optimal assay based on a specific need. No matter how appropriate and well accepted the assay is, it is recommended that a second assay using a different principle should be used to confirm the detection of cell-death. In the future, we should also have in mind that intact efferocytosis, i.e. the clearance of apoptotic cells, can promote cancer disease (Vaught et al., 2015). It is therefore important that both processes, apoptosis and efferocytosis are fine-tuned in specific tumor microenvironments. Hence, the novel functional assays for analyzing the influence of PnD on efferocytosis-mediated regulation of the tumor microenvironment needs to be developed/used.

### 2.4 Investigating perinatal derivatives actions on tumor cell migration and invasion

PnDs have been reported to both inhibit (Fong et al., 2011; Gauthaman et al., 2012; Kalamegam et al., 2018; Li et al., 2019) and induce (Kim et al., 2015; Li et al., 2015) tumor cell migration.

The spread of neoplastic disease has been described as a sequential multi-step process, termed the invasion-metastatic cascade (Martin and Jiang 2009). During migration and invasion, cells squeeze through tight interstitial spaces, which includes cellular and nuclear deformation caused by the confining microenvironment (Kramer et al., 2013). Overall, during the metastatic cascade, changes in cell-cell and cell-matrix adhesion are of paramount importance and lead to the formation of secondary tumors in distant organs and are largely responsible for the mortality and morbidity of cancer (Martin et al., 2013). There are several commonly used *in vitro* assays to investigate the effects of PnDs on tumor cell migration and invasion potential. Transwell migration and invasion assay (Boyden chamber) is the most frequently used approach. In this assay, a double chamber is filled with two media, one with an attractant (like FBS) to trigger chemotaxis. Cells are seeded in the upper well and migrate vertically between the chambers through a porous membrane (Menyhárt et al., 2016). Migrated cells can be visualized by cytological dyes or stained fluorescent and then assessed by flow cytometry, light or fluorescence microscopy, or lysed and assessed by a plate reader, usually following treatment with MTT reagent (Gauthaman et al., 2012; Kim et al., 2015; Li et al., 2015; Kalamegam et al., 2018; Yuan et al., 2018; Mandal et al., 2019; Silva et al., 2020). By coating the porous filter with ECM components like type I collagen or a basement membrane-like matrix (Matrigel (So et al., 2015; Bu et al., 2017; Meng et al., 2019)) or reduced growth factor matrix (Touboul et al., 2013), invasive cells can be detected by their ability to degrade the matrix and move through the membrane to the bottom well. Parallel measurements with ECM-coated and non-coated assays allow one to calculate an “invasive index”: the rate of invasiveness versus migration (Marshall 2011), however this approach is currently missing in *vitro* assays investigating migration and invasion potential of PnDs. The most frequently used method due to its easy setup is the transwell migration assay. More sophisticated migration assays using microfluidic migration devices overcome the limitations of traditional migration assays and promote a stable diffusion-generated concentration gradient that is consistently linear and lasts for more than 48 h. These devices are usually plastic with high optical qualities similar to those of glass, and are specially designed for video microscopy assays. At specific time intervals, images of the observation area can be acquired, allowing real-time monitoring and quantitative measurements of cell migration and thus could also be used in investigating PnDs actions on cancer cell migration and invasion.

Investigation into the effect of PnDs on the invasive potential of cancer cells has been also performed using intact or decellularized human amniotic membrane or amniochorionic membrane. These were used in some studies as a natural 3D scaffold to evaluate tumor cell metastatic and invasion potential in 3D conditions (Ganjibakhsh et al., 2019) or direct influence of PnDs on metastatic and invasion behavior of tumor cells (Touboul et al., 2013; Ramuta T. Z. et al., 2020).

### 2.5 Investigating perinatal derivatives actions on the tumor vasculature and angiogenesis

PnDs have been widely reported to produce angiogenic factors and induce angiogenesis (Bajetto et al., 2017; Dabrowski et al., 2017; Komaki et al., 2017; Wu et al., 2022), yet some studies have described antiangiogenic effects (Faraj, Stewart et al., 2015), and PnD preparation seems to be a critical point that can influence this feature (Wolbank et al., 2009).

Quantitative real time polymerase chain reaction is often performed to detect and quantify the relative expression levels of angiogenic genes, such as VEGF (Lin D et al., 2017; Mandal et al., 2019), ANG (Mandal et al., 2019; Yuan L et al., 2019), PDGF (Yuan Z et al., 2019), FGF-2 (Subramanian et al., 2012), etc. However, this method is time and resource consuming, requires subsequent post-PCR analysis and may provide only limited information on gene expression that must be followed with immunoblot or ELISA. Indeed, the latter have been widely used to detect known angiogenesis activators by immunoblot or ELISA (Table 1). However, Western blot can produce false-positive/negative results in the sample of interest and requires a larger amount of starting material. Immunophenotypic analysis to measure the expression of major angiogenic proteins, such as VEGF (Borghesi et al., 2020) can be performed with flow cytometry. Despite its ability to identify small populations and quantify the intensity of fluorescence, it still requires complex instrumentation, and highly trained technical staff to manage microfluidics, laser calibration and cleaning, as well as ample experience with the relevant software. In addition, direct and indirect immunocytochemistry (ICCH) can visualize and localize the target VEGF protein expression at a cell compartment level (Subramanian et al., 2012). Indirect ICCH can, however, be more laborious and time-consuming, with the additional risk of non-specific binding of the secondary antibody.

**TABLE 1:** Angiogenesis activators produced by PnDs.

Activator	PnDs
VEGF	Placenta-based somatic stem cells (Zhang et al., 2015)
	Placental-derived adherent stromal cells (Allen et al., 2018)
	Umbilical cord MSCs (Bajetto et al., 2017; Lin D et al., 2017; Ciavarella et al., 2015)
	Wharton jelly MSCs (Kalamegam et al., 2019; Mandal et al., 2019; Subramanian et al., 2012) Umbilical cord MSCs (Dabrowski FA et al., 2017); Amniotic membrane MSCs (Dabrowski FA et al., 2017)
FGFS	Umbilical cord MSCs (Ciavarella et al., 2015)
	Wharton jelly stem cells (Kalamegam et al., 2019)
MMPs	Placenta derived MSCs (Choi et al., 2016)
	Placental-derived adherent stromal cells (Allen et al., 2018)
TIMPs	Umbilical cord MSCs (Ciavarella et al., 2015)
	Human amniotic membrane (Modaresifar et al., 2017)
ANG	Umbilical cord MSCs (Bajetto et al., 2017)
EGF	Umbilical cord MSCs (Ciavarella et al., 2015; Dabrowski FA et al., 2017); Amniotic membrane MSCs (Dabrowski FA et al., 2017)

Monitoring the proliferation of human umbilical vein endothelial cells (HUVECs) is often used to evaluate the developing tumor vasculature. These assays have been used to estimate antiangiogenic properties of PnDs in the context of early tumor pre-vasculature. CCK8 (Yuan L et al., 2019) assay offer reproducible and easy setups and provide quantifiable data on the inhibition of endothelial cell proliferation. Alternative methods, such as Trypan blue analysis (Chen et al., 2012) might be more difficult to reproduce and validate. Monitoring of electrical impedance changes (Grzywocz et al., 2018) caused by the proliferation of HUVECs could be enabled by real-time systems. In addition, the digital endothelial tube formation assay-derived images could potentially enable the calculation of digital angiogenic indices (Alshareeda et al., 2018), covering the numerical values of tube morphometry, such as extremities, number of segments, branches and the length of tubes.

To evaluate angiogenesis *in vitro*, the rat aorta ring assay (Modaresifar et al., 2017) can be performed with PnDs. In this assay, aorta rings are dissected from the descending thoracic aorta, rinsed, and cut into circular sections of several millimeters thick. These sections are put on cultured cells and the angiogenic potential is determined by microscopically visualizing endothelial cell sprouting, polarization, and outgrowth to the periphery. This test enables evaluation of angiogenic and antiangiogenic effects of PnDs and is more representative of *in vivo* angiogenesis than two dimensional assays.

## 3 Antimicrobial effects of perinatal derivatives

One of the most intriguing characteristics of PnDs is their direct and indirect antimicrobial properties, which have therapeutic potential. PnDs have been shown to possess antimicrobial activity against various microorganisms in planktonic form and also in complex microenvironments. While the precise nature of antimicrobial action of PnDs is not well understood, it is clear that more than one mechanism working simultaneously to inhibit microbe growth and endotoxin activity, contributes to this activity (Magatti, Vertua et al., 2017). Antimicrobial properties of PnDs have been investigated in bacteria (Talmi et al., 1991; Mao et al., 2016; Mao et al., 2017; Tehrani et al., 2017; Mao et al., 2018; Ashraf et al., 2019; Palanker et al., 2019; Šket et al., 2019; Ramuta et al., 2020a), fungi (Wang, Xie et al., 2012), bacteria-infected cell cultures (Ramuta et al., 2021b) and rat *in vivo* models (Robson and Krizek 1973; Yadav et al., 2017). Antimicrobial peptides, such as α and β defensins, human cathelicidin LL37, lipocalin, elafin and secretory leukocyte protease inhibitor (SLPI), have been identified in various PnDs (King et al., 2007a; King et al., 2007b; Ramuta T.Ž. et al., 2021; Dubus et al., 2022). Furthermore, it was shown that histones H2A and H2B could also exert an antimicrobial action as a endotoxin-neutralizing barrier (Kim et al., 2002). Moreover, it was reported that hemoglobin-derived peptides purified from a human placenta exhibited antimicrobial activity. These peptides inhibited the growth of Gram-positive and Gram-negative bacteria and yeasts in micromolar concentrations, as well they reduced endotoxin activity by binding to LPS (Liepke et al., 2003; Dubus et al., 2022). In case of decellularized Wharton’s jelly tissue, the mass spectrometry analysis showed the release of antimicrobial molecules involved in the innate immune response but also some molecules involved in bacterial agglutination such as fibrinogen beta chain and Fibulin 1 (Dubus et al., 2022). These molecules are thought to exert a bacteriostatic effect on both Gram-positive and Gram-negative strain. In this section, we offer an overview of the most common *in vitro* and *in vivo* functional assays that have been used for evaluating the antimicrobial activity of PnDs against bacteria and fungi.

The gold standards for antimicrobial susceptibility testing have been set by the Clinical and Laboratory Standards Institute, however, due to the versatility of PnDs, the following assays do not strictly follow the standard protocols, as they had to be adapted to enable the analysis of various PnDs-derived preparations in bacterial suspensions.

### 3.1 Investigating the antimicrobial effects of perinatal derivatives in bacterial suspension

A broth (micro)dilution assay is a simple and inexpensive method which is consequently often used to test the susceptibility of bacterial isolates to various antimicrobials. PnDs (often at various dilutions) are added to liquid broth media, which are then inoculated with bacterial suspensions. Following incubation (the length of which can vary from a couple of hours to several days), bacterial growth is evaluated based on turbidity by using visual or spectrophotometric methods (Jorgensen and Ferraro 2009; Sung et al., 2016; Šket et al., 2019; Dubus et al., 2020; El-Mahdy et al., 2021). Furthermore, bacterial growth can be also quantified by plating serial dilutions of bacterial suspensions incubated with the antimicrobial agent and counting the colony forming units (CFU) (Thadepalli et al., 1977; Mao et al., 2017; Šket et al., 2019). The broth microdilution method is often used to determine the minimum inhibitory concentration (Wiegand et al., 2008; Kim et al., 2012; Yadav et al., 2017) of an antimicrobial agent, which is the lowest concentration that will inhibit the visible growth of a microorganism after overnight incubation. The advantages of using the broth (micro)dilution test are the generation of a quantitative result and high reproducibility, while the main shortcoming is that it is less sensitive and more time-consuming (Jorgensen and Ferraro 2009) than some of the other functional assays for determination of the antimicrobial effects of PnDs. Furthermore, the presence of dead bacteria in the presence of PnDs cannot be determined.

A similar method is a disk diffusion assay. Namely, a bacterial inoculum is plated onto the surface of the agar plate and subsequently PnDs (often at various dilutions) are applied to the inoculated agar surface. After incubation of the agar plates the inhibition zones around the site of application of the PnDs antimicrobials are measured (Talmi et al., 1991; Kjaergaard et al., 2001; Jorgensen and Ferraro 2009; Tehrani et al., 2013; Tehrani et al., 2017; Šket et al., 2019; Ramuta et al., 2020b; Ramuta T.Ž. et al., 2021). The shortcoming of this method is its inability to precisely determine the minimum inhibitory concentration of the antimicrobial agent.

### 3.2 Investigating the antimicrobial effects of perinatal derivatives in complex(micro)environments

The antimicrobial properties of PnDs have been evaluated in complex (micro)environments, such as biofilms and bacteria-infected epithelia. The effect of PnDs on biofilms has been evaluated by the biofilm formation assay (Dubus et al., 2020; El-Mahdy et al., 2021). The antiadhesive and antifouling properties of PnDs such as Wharton’s jelly were mainly attributed the presence of hyaluronic acid and its composites (Drago et al., 2014; Marcuzzo et al., 2017). Biofilm is defined as a bacterial community which is metabolically heterogeneous and embedded in a self-produced extracellular matrix, causing a critical virulence factor responsible for treatment failure and chronicity in medical device-related infections. The (micro)plates are inoculated with fresh bacterial cell suspensions and subsequently the PnDs are added. The biofilms are grown for several hours to days. After staining with crystal violet, the effect of the PnDs on biofilm is quantified spectrophotometrically. This is an inexpensive and easy method which can directly evaluate the biofilm formation on several surfaces (i.e., titanium alloys, calcium phosphate, polymers, etc). However, there are a few shortcomings of the biofilm formation assay. Firstly, it is not possible to distinguish whether the antimicrobial effect can be attributed to killing of planktonic bacteria before the biofilm forms or to the specific antibiofilm effects, because the antimicrobial agent is added to the bacterial suspension before the biofilm is formed. Furthermore, the crystal violet stains the whole biomass (bacteria and exopolymers) and not only the living bacteria, hence it is not possible to determine the ratio of alive vs. dead bacteria in the biofilm (Haney et al., 2018). To get comprehensive insight into how PnDs affect biofilms, additional experiments should be performed also on pre-formed biofilms (Segev-Zarko and Shai 2017). To determine the metabolically active cells in the biofilm, resazurin staining (Yadav et al., 2017) is used. To evaluate the viability of the cells, forming the biofilm, the live/dead bacterial viability kit (El-Mahdy et al., 2021; Dubus et al., 2022) is used. To evaluate the morphology of the biofilm, most frequently confocal microscopy or scanning electron microscopy are used (Yadav et al., 2017; Mao et al., 2018; El-Mahdy et al., 2021).

To evaluate the antimicrobial effect of PnDs in bacteria-infected epithelia, the following cellular *in vitro* models have been established. Cells are grown to confluence using antibiotic-free medium and then inoculated with bacteria. The number of viable bacteria in culture medium are quantified by plating serial dilutions of bacterial suspensions and counting the CFU (Josse et al., 2014; Sung et al., 2016; Dubus et al., 2020; Ramuta et al., 2021a; El-Mahdy et al., 2021). Next, the permeabilization agent is used to release the intracellular bacteria, which are then quantified by plating serial dilutions of bacterial suspensions and counting the CFU. (Josse et al., 2014; Dubus et al., 2020; El-Mahdy et al., 2021). Moreover, the effect of bacteria and PnDs on eukaryotic cells are evaluated by quantifying the number and viability of eukaryotic cells and also the intracellular localization of bacteria is assessed by using various methods of light, confocal and electron microscopy (Josse et al., 2014; Ramuta et al., 2021b; El-Mahdy et al., 2021). To gain better understanding of the effect of PnDs in bacteria-infected epithelia, special attention must be given to establishment of physiologically-relevant *in vitro* models. For example, a multilayered biomimetic porcine urothelial model that has been shown to react differently to pathogenic vs. non-pathogenic *E. coli* strains has been used for evaluating the antimicrobial properties of PnDs (Ramuta T.Ž. et al., 2021; Predojević et al., 2022). Similar complex *in vitro* models have been established to study the host-pathogen interactions in the airway mucosa (Marrazzo et al., 2016; Hasan et al., 2018), intestine (Pearce et al., 2018; García-Díaz et al., 2022) and skin (Bolle et al., 2020), but have not yet been used for evaluation of PnDs.

The antimicrobial properties of PnDs have also been evaluated *in vivo* using several different methods. These studies have assessed the antimicrobial activity by quantifying bacteria in the spleen or blood or indirectly by measuring antimicrobial cytokines. For example, PnDs have been shown to protect against experimental sepsis (murine cecal ligation and puncture model of sepsis) (Parolini et al., 2014; Laroye et al., 2019) and *E. coli*-induced acute lung injury (Sung et al., 2016).

## Conclusion

PnDs have demonstrated contradictory effects in the field of oncology. Various factors that include the specific PnD tissue of origin, the type and size of tumor, the PnD injection route, the treatment regimen and interactions with the host appear to play a role in determining whether PnD exert pro-tumorigenic or antitumorigenic properties. To facilitate the translation of PnDs towards the clinic, it is crucial to standardize procedures for evaluating the properties of PnDs and to define the criteria that distinguish each PnDs as suitable for clinical use. PnDs-derived preparations are a very versatile group, ranging from cells and their conditioned media to tissue-derived scaffolds. This must be taken into account when selecting methods and defining criteria for validating the multimodal functions of PnDs to be used in oncological and antimicrobial applications. Another challenge is the development of assays that can efficiently and reproducibly measure the anticancer and antimicrobial properties of PnDs in *vitro* and *in vivo* models of cancer and infection.

In summary, there is a growing awareness that PnDs possess precisely tuned anticancer and antimicrobial activities. In this review, we therefore present the most commonly used functional assays (Table 2) with their advantages and disadvantages in assessing the anticancer and antimicrobial functionality of PnDs. This must be considered in future research and in the development of more effective PnDs therapies.

**TABLE 2:** Summary of assays used to detect antitumor and antimicrobial effects of PnDs.

Anti-microbial effects	Functional assays		References
Tumor cell proliferation and metabolism	Incorporation of radiolabeled DNA precursor 3H-thymidine into the new strands of chromosomal DNA Incorporation of a synthetic nucleoside analog BrdU or EdU into the new strands of chromosomal DNA Immunolabeling of cell cycle-related proteins (e.g., Ki67, cyclins) Colony forming unit (CFU) assay		Ayuzawa et al. (2009) Magatti et al. (2012) Marleau et al. (2012) Yuan et al. (2013) Riedel et al. (2019) Ramuta et al. (2020c) Tian et al. (2010) Gauthaman et al. (2012) Han et al. (2014) Hendijani et al. (2015) Wang M et al. (2015) Janev et al. (2021) Lin et al. (2014) Riedel et al. (2019) Magatti et al. (2012) Ayuzawa et al. (2009) Liu et al. (2013) Ciavarella et al. (2015) Li et al. (2015) Wang M et al. (2015) Wang W et al. (2015)
	Colorimetric assays for metabolism analysis	MTT assay Cell Counting Kit-8	Li et al. (2011) Chao et al. (2012) Gauthaman et al. (2012) Kang et al. (2012) Liu et al. (2013) Lin et al. (2014) Niknejad et al. (2014) Rolfo et al. (2014) Bonomi et al. (2015) Lang et al. (2015) Li et al. (2015) Kamalabadi-Farahani et al. (2018) Mandal et al. (2019) Riedel et al. (2019) Wu et al. (2013) Yan et al. (2013) Yang et al. (2014a) Kim et al. (2015) Di Germanio et al. (2016)
	Annexin V/PI assay and flow cytometry		Gauthaman et al. (2012) Wu et al. (2013) Niknejad et al. (2014) Yang et al. (2014b) Mamede et al. (2015) Lin et al. (2016) Paris et al. (2016) Shen et al. (2016) Lin D et al. (2017) Chai et al. (2018) Jiao et al. (2018) Yuan et al. (2018) Khalil et al. (2019) Rezaei-Tazangi et al. (2020) Silva et al. (2020)
Tumor cell death	TUNEL assay		Wu et al. (2013) Niknejad et al. (2014)
	Detection of apoptosis regulators (e.g., caspases, Bcl-2, DAMPs)	Flow cytometry Multi-detection plate reader Immunoblot Immunohistochemistry, Immunofluorescence RT-PCR	Rezaei-Tazangi et al. (2020) Wu et al. (2013) Lin et al. (2016) Kalamegam et al. (2018) Mandal et al. (2019) Mamede et al. (2015) Dzobo et al. (2016) Shen et al. (2016) Yuan et al. (2018) Dzobo et al. (2016) Lin H et al. (2017) Yang et al. (2014a)
	
	Evaluation of mitochondrial membrane potential (MMP)		Mamede et al. (2015) Lin et al. (2016)
	Evaluation of the mitochondrial status by membrane permeable dye JC-1		Faridvand et al. (2019)
	Evaluation of the activity of the entire electron transfer system		Banerjee et al. (2015) Poženel et al. (2019) Banerjee et al. (2018b)
Tumor cell migration and invasion potential	Transwell migration and invasion assay (Boyden chamber)		Gauthaman et al. (2012) Kim et al. (2015) Li et al. (2015) Kalamegam et al. (2018) Yuan et al. (2018) Mandal et al. (2019) Silva et al. (2020)
	Evaluation of the metastatic and invasion potential of cancer cells on intact or decellularized human amniotic membrane- or amniochorionic membrane-derived scaffolds		Ganjibakhsh et al. (2019) Touboul et al. (2013) Ramuta et al. (2020a)
Angiogenesis	Quantitative RT-PCR for detection of angiogenic genes (e.g., VEGF, ANG, PDGF)		Lin D et al. (2017) Mandal et al. (2019) Yuan L et al. (2019) Subramanian et al. (2012)
	Immunodetection of factors affecting tumor cell angiogenesis	Immunoblot, ELISA Flow cytometry Immunocytochemistry	Zhang et al. (2015) Allen et al. (2018) Bajetto et al. (2017) Lin H et al. (2017) Ciavarella et al. (2015) Kalamegam et al. (2019) Mandal et al. (2019) Subramanian et al. (2012) Choi et al. (2016) Modaresifar et al. (2017) Bajetto et al. (2017) Dabrowski FA et al. (2017) Borghesi et al. (2020) Subramanian et al. (2012)
	Monitoring proliferation of human umbilical vein endothelial cells	Cell Counting Kit-8 Trypan blue assay Monitoring of electrical impedance changes	Yuan Z et al. (2019) Chen et al. (2012) Grzywocz et al. (2018)
*In vivo* assessment of tumor response	Rat aorta ring assay Measurements of tumor diameters Optical imaging techniques		Modaresifar et al. (2017) Du et al. (2014) Ma et al. (2015) Bu et al. (2017) Yuan L et al. (2019) Di et al. (2014) Leng et al. (2014) Ciavarella et al. (2015) Cafforio et al. (2017) Zhang et al. (2017) Yuan Z et al. (2019) Chetty et al. (2020)
Bacterial growth	Broth (micro)dilution assay Disk diffusion assay		Jorgensen and Ferraro (2009) Sung et al. (2016) Dubus et al. (2020) El-Mahdy et al. (2021) Thadepalli et al. (1977) Mao et al. (2017) Šket et al. (2019) Wiegand et al. (2008) Kim et al. (2012) Yadav et al. (2017) Kjaergaard et al. (2001) Talmi et al. (1991) Jorgensen and Ferraro, (2009) Tehrani et al. (2013) Tehrani et al. (2017) Šket et al. (2019) Ramuta et al. (2021a) Ramuta et al. (2020b)
Evaluation of biofilms	Biofilm formation assay Determination of metabolically active cells by resazurin staining Live/dead bacterial viability Morphology evaluation by light and electron microscopy		Dubus et al. (2020) El-Mahdy et al. (2021) Yadav et al. (2017) El-Mahdy et al. (2021) Dubus et al. (2022) Yadav et al. (2017) Mao, et al. (2018) El-Mahdy et al. (2021)
Evaluation of antimicrobial activity in a complex (cellular) microenvironment			Josse et al. (2014) Sung et al. (2016) Dubus et al. (2020) El-Mahdy et al. (2021) Ramuta et al. (2021c)
